# *Il-1r1* drives leukemogenesis induced by *Tet2* loss

**DOI:** 10.1038/s41375-022-01665-3

**Published:** 2022-08-12

**Authors:** Sarah S. Burns, Ramesh Kumar, Santhosh Kumar Pasupuleti, Kaman So, Chi Zhang, Reuben Kapur

**Affiliations:** 1 Medical Scientist Training Program, Indiana University-Purdue University, Indianapolis, USA; 2 Medical and Molecular Genetics Graduate Program, Indiana University-Purdue University, Indianapolis, USA; 3 The Herman B. Wells Center for Pediatric Research, Indiana University-Purdue University, Indianapolis, USA; 4 Department of Pediatrics, Indiana University-Purdue University, Indianapolis, USA; 5 Department of Biostatistics and Health Data Science, Indiana University-Purdue University, Indianapolis, USA; 6 Center for Computational Biology and Bioinformatics, Indiana University School of Medicine, Indiana, USA; 7 Molecular Biology and Biochemistry, Indiana University School of Medicine, Indiana, USA; 8 Medical and Molecular Genetics, Indiana University School of Medicine, Indiana, USA; 9 Microbiology and Immunology, Indiana University School of Medicine, Indiana, USA

**Keywords:** Haematopoietic stem cells, Cancer models

Loss of the *ten-eleven translocation methylcytosine dioxygenase 2* (*Tet2*) gene, which is commonly mutated in hematological malignancies, dysregulates inflammatory pathways, including the interleukin-1 (IL-1) pathway [1–3]. Roles for IL-1 signaling have been reported in terminally differentiated hematopoietic cells and in non-cell autonomous contexts [3, 4]. However, our group demonstrated that inhibition of inflammatory pathways can suppress clonal hematopoiesis (CH), indicating potential direct roles for hematopoietic stem and progenitor cells (HSPCs) in inflammation [5]. As *TET2* mutations are often present in HSPCs and provide these cells with a competitive advantage, dysregulation of the IL-1 pathway in HSPCs may contribute to leukemogenesis and may catalyze the progression of preleukemic states to malignancy [6].

Mutations in the *TET2* gene are detected in a variety of myeloid malignancies, including acute myeloid leukemia (AML) [6]. Similarly, *Tet2*^−*/*−^ transgenic mice and recipient mice transplanted with *Tet2*^−*/*−^ bone marrow (BM) exhibit splenomegaly, monocytosis, extramedullary hematopoiesis, and expansion of the Lin^-^;Sca1^+^;c-Kit^+^ (LSK) population [7]. Acute and chronic IL-1 exposure expands myeloid cells at the expense of lymphoid cells; however, chronic exposure ultimately depletes the ability of hematopoietic stem cells (HSCs) to self-renew [8]. While previous studies have investigated the exogenous effects of IL-1α and IL-1β on hematopoiesis and on mature hematopoietic cells, IL-1R1, the primary of ten IL-1 receptors, binds to multiple proteins, including IL-1α, IL-1β, IL-1 receptor antagonist, IL-38, and its co-receptor IL-1 receptor accessory protein, underscoring that the full spectrum of the consequences of IL-1R1-dependent signaling in HSPCs is not yet known [9].

Based on these findings, we hypothesized that loss of the *Il-1r1* gene would rescue the hematological abnormalities associated with *Tet2* deficiency at the HSPC level. Both *Il-1r1* and *Tet2* are expressed in multiple hematopoietic cell types, including high expression in HSPCs (Supplementary Fig. 3A, B) [10]. To determine whether loss of *Il-1r1* can ameliorate malignancy, we generated *Tet2*^−*/*−^*;Il-1r1*^−*/*−^ mice and analyzed peripheral blood (PB) counts in a large cohort. The frequencies of myeloid cells were elevated in *Tet2*^−*/*−^ mice; however, these cell types were restored to wild-type (WT) levels in *Tet2*^−*/*−^*;Il-1r1*^−*/*−^ mice (Fig. 1A). In addition to an increase in myeloid cells, lymphocyte frequency was reduced in *Tet2*^−*/*−^ mice, demonstrating a myeloid shift at the expense of lymphocytes (Fig. 1A). Higher red cell distribution width (RDW-CV) was recently reported as a measure of pro-inflammatory states and correlated with an increased risk of AML in humans [11]. Consistent with a pro-inflammatory state due to *Tet2* loss, RDW-CV was increased in *Tet2*^−*/*−^ mice and was relieved by inactivation of *Il-1r1* (Fig. 1B). In representative mice from this larger cohort, *Tet2*^−*/*−^ mice had larger spleen sizes and weights, which were corrected by loss of *Il-1r1* (Fig. 1C and Supplementary Fig. 1A). These mice showed similar alleviations of elevated myeloid frequencies and suppressed lymphocyte frequencies, supporting a role for *Il-1r1* at the stem-cell level (Supplementary Fig. 1B–D). To examine this possibility, the levels of HSPCs were measured by flow cytometry. Elevated levels of LSKs, long-term HSCs (LT-HSCs), short-term HSCs (ST-HSCs), multipotent progenitor (MPP) pools 2, 3/4, 3, and 4, and common myeloid progenitors (CMPs) were detected in *Tet2*^−*/*−^ mice, and these increases were rescued in *Tet2*^−*/*−^*;Il-1r1*^−*/*−^ mice, suggesting that loss of *Il-1r1* rescues the expansion of HSPCs associated with *Tet2* deficiency (Fig. 1D; Supplementary Fig. 1E–K). Comparable to other progenitor populations, Lin^-^;Sca1^+^ cells, which represent a subset of lymphoid progenitors that differ from common lymphoid progenitors (CLPs), were also increased in *Tet2*^−*/*−^ mice (Fig. 1D) [12]. However, Lin^-^;CD127^+^ progenitors within this Lin^-^;Sca1^+^ population were suppressed, indicating the presence of a block in lymphopoiesis at this stage (Fig. 1D). The increases in LSKs, LT-HSCs, ST-HSCs, and MPPs may represent a compensatory response to this blockage. *Il-1r1* inactivation relieved inhibition of Lin^-^;CD127^+^ cells and normalized the levels of mature lymphoid cell types (Fig. 1D). These findings show that *Il-1r1* loss can rescue *Tet2*-associated HSPC abnormalities. Together, they support roles for IL-1R-dependent signaling at the level of HSPCs, in the correction of myeloid disease, in the modulation of the pro-inflammatory state associated with *Tet2* deficiency, and in the balance of myeloid and lymphoid cell types.

**Fig. 1:**
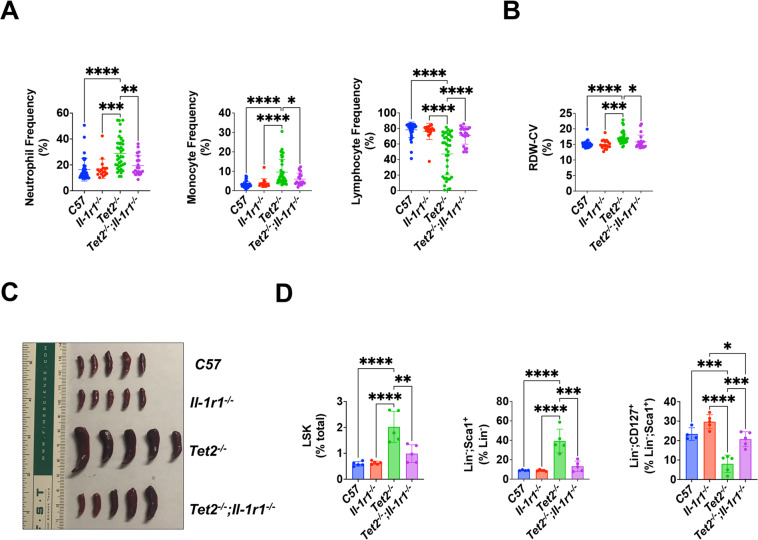
*Tet2*^−*/*−^*;Il-1r1*^−*/*−^ mice demonstrated a correction of myeloid cell elevation, lymphocyte suppression, RDW-CV, spleen size, and HSPC levels. **A**, **B** Means for neutrophil, monocyte, and lymphocyte frequencies and RDW-CV are displayed for a large cohort of mice over a range of ages. *n* = 17–38 per group. **C** Gross photographs of spleens for representative mice are presented. **D** Mean frequencies for LSK, Lin^−^;Sca1^+^, and Lin^−^;CD127^+^ cells are shown for representative mice. *p ≤ 0.05, **p ≤ 0.01, ***p ≤ 0.001, ****p ≤ 0.0001. Error bars represent standard deviation. *n* = 4−5 per group.

To investigate whether *Il-1r1* deficiency rescues *Tet2*-associated hematological malignancies in a cell autonomous manner, we performed a competitive transplantation of BM containing HSPCs from C57 (CD45.2), Boy/J (CD45.1), *Tet2*^−*/*−^ (CD45.2), *Il-1r1*^−*/*−^ (CD45.2), and *Tet2*^−*/*−^*;Il-1r1*^−*/*−^ (CD45.2) donor mice into CD45.1- and CD45.2-expressing F1 recipient mice and evaluated the effects of *Il-1r1* loss on engraftment and on *Tet2*^−*/*−^ HSPCs and mature hematopoietic cell types (Supplementary Fig. 2A). Inactivation of *Il-1r1* reduced the increased engraftment of CD45.2-expressing cells and the high numbers and frequencies of white blood cells (WBC) and myeloid cells detected in mice transplanted with *Tet2*^−*/*−^ BM (Fig. 2A; Supplementary Figs. 2B–G and 4A, B). Similar corrections of myeloid cells were observed in PB smears and Gr-1^+^ myeloid cells (Fig. 2A, B). As in the transgenic mice, loss of *Il-1r1* corrected spleen sizes and weights (Fig. 2C; Supplementary Fig. 2H). Consistent with relief of lymphocyte suppression, an increased lymphocyte frequency and elevated percentages of CD4^+^ T cells, CD8^+^ T cells, natural killer (NK) cells, and plasmacytoid dendritic cells (pDCs), all cells of lymphoid origin, were detected in mice transplanted with *Tet2*^−*/*−^*;Il-1r1*^−*/*−^ BM, demonstrating that loss of *Il-1r1* at the HSPC level can restore the levels of multiple lymphoid cell types (Supplementary Fig. 4C–N). Similar to the transgenic mice, increased levels of LSK, MPP2, and MPP3/4 cells associated with *Tet2* deficiency were rescued in mice transplanted with *Tet2*^−*/*−^*;Il-1r1*^−*/*−^ BM, corroborating a role for *Il-1r1* in the regulation of cell populations that contain leukemia-initiating cells (Supplementary Fig. 3C–E). In addition, in mice transplanted with *Tet2*^−*/*−^ BM, Lin^-^;c-Kit^+^ cells were reduced, while Lin^-^;Sca1^+^ cells were significantly elevated (Supplementary Fig. 3F, G). These changes were reversed in mice transplanted with *Tet2*^−*/*−^*;Il-1r1*^−*/*−^ BM, further supporting profound shifts in myeloid and lymphoid populations (Supplementary Fig. 3F, G). Collectively, these findings suggest that *Il-1r1* loss abrogates hematological malignancy and corrects disruption of the myeloid-lymphoid balance via cell autonomous mechanisms in HSPCs.

**Fig. 2:**
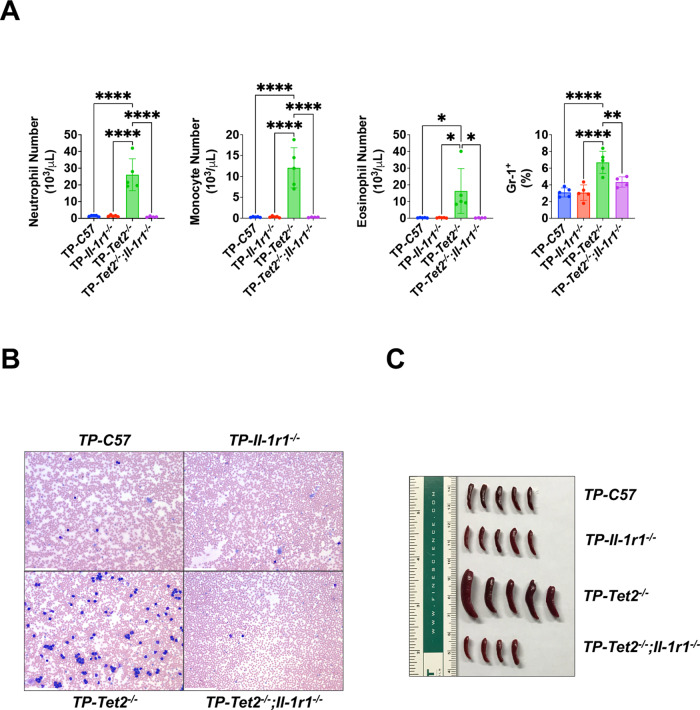
Recipients of *Tet2*^−*/*−^*;Il-1r1*^−*/*−^ BM exhibited rescue of increased levels of myeloid cells and spleen size. **A** Mean absolute counts of neutrophils, monocytes, and eosinophils and the frequency of Gr-1^+^ myeloid cells at six-months post-transplant. **B** Photographs of representative blood smears are shown and were acquired at 20X magnification. The scale bar denotes 20 μm. **C** Gross photographs of spleens are presented. *p ≤ 0.05, **p ≤ 0.01, ***p ≤ 0.001, ****p ≤ 0.0001. Error bars represent standard deviation. *n* = 4–5 per group.

To investigate whether inactivation of IL-1 signaling in BM cells alleviates systemic inflammation associated with *Tet2* deficiency, serum cytokine levels were measured. Consistent with previous studies, loss of *Tet2* led to increases in multiple cytokines and chemokines, including tumor necrosis factor α (TNFα) and the interferon-γ (IFN-γ)-inducible genes IFN-γ-induced protein 10 (IP-10/CXCL10) and monokine induced by IFN-γ (MIG/CXCL9) (Supplementary Fig. 5A–C) [1, 2]. These cytokines and chemokines were restored to WT levels in mice transplanted with *Tet2*^−*/*−^*;Il-1r1*^−*/*−^ BM (Supplementary Fig. 5A–C). TNFα promotes the expansion of *Tet2*^−*/*−^ cells in vitro, indicating a non-cell autonomous role [2]. However, we demonstrated that TNFα levels were also elevated in mice transplanted with *Tet2*^−*/*−^ BM and that this increase was rescued by *Il-1r1* loss in a cell autonomous manner. TNFα and IFNγ can control the levels of Lin^-^;Sca1^+^ cells and Sca1 expression and can promote myeloid expansion and regeneration, providing opportunities for antagonistic regulation of lymphoid and myeloid populations [13–15]. Together, these results support roles for IL-1 signaling in HSPCs in modulating the myeloid-lymphoid balance and in determining the pro-inflammatory status of *Tet2*^−*/*−^ mature hematopoietic cells. Based on these findings, we propose a mechanism by which loss of *Tet2* leads to a pro-inflammatory state that is characterized by high levels of TNFα and IFN-γ and that causes a myeloid bias at the expense of lymphoid cells (Supplementary Fig. 2I). This shift was evidenced by the elevation of CMPs and the suppression of Lin^-^;CD127^+^ lymphoid progenitors in *Tet2*-deficient contexts. The loss of IL-1R1-dependent signaling rescued these disruptions in normal hematopoiesis, abrogating myeloid disease and bolstering its potential as a therapeutic target (Supplementary Fig. 2J).

To determine the clinical relevance of *IL-1R1* expression in patients with myeloid malignancies, we examined two publically-available datasets for *IL-1R1* expression levels and correlated these levels with survival. Both pediatric and adult AML patients with higher levels of *IL-1R1* expression exhibited decreased survival, suggesting a role for *IL-1R1* in AML pathogenesis (Supplementary Fig. 6A, B). To evaluate whether the effects of *IL-1R1* on survival are specific to distinct AML subtypes, survival was analyzed in the context of high and low *IL-1R1* expression in ten adult AML subtypes. High expression of *IL-1R1* conferred reduced survival in subtypes containing mutations in the *CBFB-MYH11*, *NPM1*, or *p53C* genes (Supplementary Fig. 7A–K). IL-1 signaling has been implicated in the expansion of CD34^+^ human AML cells, further supporting its clinical relevance [4]. These results underscore the potential therapeutic implications of IL-1R-dependent signaling in myeloid malignancies and suggest that patient stratification may be needed.

In summary, we have shown that loss of *Il-1r1* in *Tet2*^−*/*−^ HSPCs rescued several abnormalities associated with *Tet2* deficiency, including the elevation of LSK cells, the pro-inflammatory state, and the myeloid-lymphoid imbalance. Furthermore, high expression of *IL-1R1* had a clinically significant impact on AML survival. Collectively, these findings underscore a potential therapeutic role for IL-1 signaling in the myeloid aspects of hematological malignancies and preleukemic conditions at the stem-cell level.

## Supplementary information

Supplementary Information

## Data Availability

The *IL-1R1* expression data are publically available. Other data generated in this study are available from the corresponding author on reasonable request.
