# Supplementary material for: Il-1r1 drives leukemogenesis induced by Tet2 loss
Source: Leukemia. 2022 Aug 12;36(10):2531–4. doi: 10.1038/s41375-022-01665-3 (PMC9522579; doi:10.1038/s41375-022-01665-3)
Supplement: Supplementary file 1 — Supplementary Information [file 41375_2022_1665_MOESM1_ESM.pdf]

Supplementary Materials and Methods

Analysis of IL-1R1 expression in AML patients

Gene expression data from two AML datasets was retrieved from the public domain, including Therapeutically Applicable Research To Generate Effective Treatments (TARGET) AML data from the Genomic Data Commons (GDC) data portal and GSE37642 from the Gene Expression Omnibus (GEO) database. In each dataset, we split the samples into two groups, namely the top 50% expressing higher levels of *IL-1R1* and the bottom 50% expressing lower levels of *IL-1R1*. log(FPKM+1) normalized gene expression level was used for the TARGET data, and log(RMA) normalized gene expression level was analyzed for the GSE37642 data. We utilized a log-rank test to evaluate the difference in prognosis of the samples in these two groups. To evaluate the effect of *IL-1R1* expression on the survival of AML subtypes, we identified ten AML subtypes in the GSE37642 dataset using subtype-specific genes by gene expression stratification (https://www.meb.ki.se/shiny/truvu//AMLSubtypeSpecificDiscovery/). The top 100-1000 genes for each subtype were used in this analysis. Subtype hierarchical cluster analysis and survival analysis were performed for the samples in each subtype.

Mice and genotyping

C57 and Boy/J mice were obtained from the *In Vivo* Therapeutics Core at Indiana University Simon Cancer Center, and *Tet2*$^{-/-}$ mice were acquired from Dr. Mingjiang Xu [7]. *Tet2*$^{-/-}$;*Il-1r1*$^{-/-}$ mice were generated by breeding *Tet2*$^{-/-}$ mice with *Il-1r1*$^{-/-}$ mice (Strain number: 003245; Jackson Laboratories, Bar Harbor, Maine, USA) and then intercrossing *Tet2*$^{+/-}$;*Il-1r1*$^{+/-}$ progeny. These mice were genotyped using primers against

Tet2 WT (Forward: 5'-CCATGCAGGGAAGACAAGAGTAGC-3'; Reverse: 5'-ATCTTGTTTGGATGGAGCCCAGAG-3') and *Tet2*-deficient (Forward: 5'-CTCTGATATGAGTTACTGGGGAGG-3'; Reverse: 5'-GGATGTGCTGCAAGGCGATTAAGT-3') alleles and *Il-1r1* WT and *Il-1r1*-deficient alleles (Mutant forward: 5'-CTCGTGCTTTACGGTATCGC-3'; WT forward: 5'-GGTGCAACTTCATAGAGAGATGA-3'; Common reverse: 5'-TTCTGTGCATGCTGGAAAAC-3') [7]. In the large cohort of transgenic mice, PB counts from mice across a range of ages (three months to ten months) were used. In the representative group used for analysis of HSPCs, approximately ten-month-old mice were evaluated.

Competitive Transplantation

BM from ten-to-eleven-month-old C57/B6, *Tet2*$^{-/-}$, *Il-1r1*$^{-/-}$, and *Tet2*$^{-/-}$;*Il-1r1*$^{-/-}$ or three-month-old Boy/J mice was isolated, and 1:1 mixtures of C57;Boy/J, *Tet2*$^{-/-}$;BoyJ, *Il-1r1*$^{-/-}$;Boy/J, or *Tet2*$^{-/-}$;*Il-1r1*$^{-/-}$;Boy/J BM were prepared. Boy/J mice express CD45.1, and C57, *Tet2*$^{-/-}$, *Il-1r1*$^{-/-}$, and *Tet2*$^{-/-}$;*Il-1r1*$^{-/-}$ mice express CD45.2. Lethally-irradiated F1 mice expressing both CD45.1 and CD45.2 were transplanted with the aforementioned BM mixtures. These mice were maintained on mouse chow containing doxycycline for three to four weeks. PB counts were measured using an Element HT5 Veterinary Hematology Analyzer (Heska, Loveland, Colorado, USA). Engraftment of the transplanted cells in the PB was monitored by flow cytometric analysis using antibodies against CD45.1 and CD45.2. Six months after the transplant, mice were harvested for analysis of PB, spleen, BM, and serum cytokines. PB smears were prepared, stained with modified

Wright-Giemsa stain, and photographed using a Leica Thunder 2D Tissue semi-automated microscope system (Leica Microsystems, Inc., Deerfield, IL, USA).

Flow Cytometry

Cell suspensions were prepared from PB, spleen, and BM and were used for flow cytometric analysis. Briefly, red blood cell lysis was performed, and cell suspensions were incubated with antibodies against appropriate cell type-specific markers. To evaluate engraftment, the fluorophore-conjugated antibodies CD45.1-APC (catalog #: 110714) and CD45.2-FITC (catalog #: 109806) were used for most assays. To measure engraftment in progenitor populations, CD45.1- PerCp/Cy5.5 (catalog #: 110728) and CD45.2-FITC (catalog #: 109806) were used. Mature hematopoietic cells, including myeloid cells, B cells, and T cells, were detected using Gr-1-APC/Cy7 (catalog #: 108424), CD11b-PE (catalog #: 101208), CD3-PE/Cy7 (catalog #: 100220), and B220-PerCp/Cy5.5 (catalog #: 103236). $CD4^+$ and $CD8^+$ T cells were assessed using CD4-APC (catalog #: 100412) and CD8α-PerCP/Cy5.5 (catalog #:100734). Dendritic cells were detected using CD11b-APC/Cy7 (catalog #: 101226), CD11c-PerCP/Cy5.5 (catalog #: 117328), B220-PE (catalog #: 103208), and MHCII-BV605 (catalog #: 107639) antibodies. NK cells were evaluated using CD3e-PE/Cy7 (catalog #: 100320) and NK1.1-PE (catalog #: 108708) antibodies. To identify HSPCs in the transplanted mice, BM cell suspensions containing two million cells were stained with a Lin-PE cocktail consisting of TER-119-PE (catalog #: 116208;), Gr-1-PE (catalog #: 116208;), B220-PE (catalog #: 103208), and CD3-PE (catalog #: 100206) and the following antibodies: c-KIT-BV421 (catalog #: 135124), Sca-1-PE/Cy7 (catalog #: 108114), CD48-

APC/Cy7 (catalog #: 103432), and CD150-PerCp/Cy5.5 (catalog #: 115922). Myeloid progenitor cells in the transplanted mice were examined using the Lin-PE cocktail described above and the following antibodies: c-Kit-APC (catalog #: 135108), Sca-1-PE/Cy7 (catalog #: 108114), CD16/32-APC/Cy7 (catalog #: 101328), and CD34-BV421 (catalog #: 119321). In the transgenic mice, myeloid and lymphoid progenitor populations were assessed using a Lin-Pacific Blue cocktail (Cat#: 133310), CD16/32-PE/Cy7 (Cat#: 101318), CD127-BV605 (Cat#: 135041), CD150-PerCP/Cy5.5 (Cat#: 115922), c-Kit-APC (Cat#: 105812), CD48-FITC (Cat#: 103404), Sca1-PE (Cat#: 108108), and CD34-APC/Cy7 (Cat#: 128622). In the transgenic mice, HSPCs were evaluated using the Lin-Pacific Blue cocktail (Cat#: 133310), Flt3-PE/Cy5 (Cat#: 135312), CD150-PerCP/Cy5.5 (Cat#: 115922), c-Kit-APC/Cy7 (Cat#: 105826), CD48-FITC (Cat#: 103404), and Sca1-PE (Cat#: 108108). All antibodies were purchased from Biolegend (San Diego, California, USA). Cell populations were analyzed on a BD Canto, a BD LSR II (LSR4), or a BD Fortessa using FACSDiva software and were quantified using FlowJo software (TreeStar, Inc., Ashland, Oregon, USA).

Preparation of serum for cytokine analysis

Serum was isolated from PB. Briefly, following blood collection, the samples were incubated in uncoated tubes for 30 minutes to one hour at room temperature to allow coagulation. They were then centrifuged at 1000xg for ten minutes at 4°C. The supernatants were collected, diluted 1:1 in PBS, stored at -80°C, and submitted to Eve Technologies® (Calgary, Canada) for multiplex cytokine analysis.

Statistical Analysis

One-way ANOVA tests with post-hoc analysis were used for all comparisons. All statistical analyses were performed using Prism software (GraphPad, San Diego, California, USA).

Study Approval

All experiments involving animals were approved by the Institutional Animal Care and Use Committee at Indiana University School of Medicine.

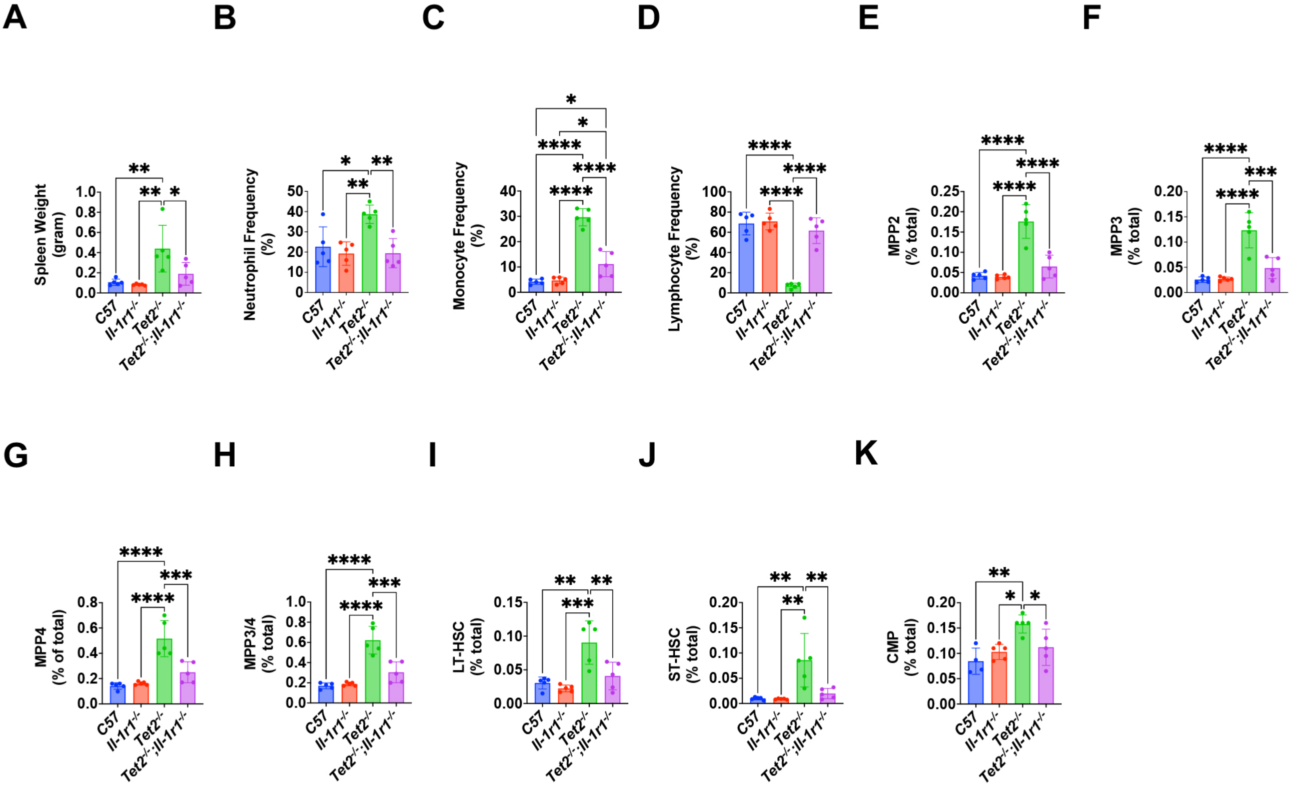

Supplementary Figure 1. Spleen weight and the frequencies of neutrophil, monocyte, lymphocyte, and HSPC populations were restored in *Tet2*$^{-/-}$;*Il-1r1*$^{-/-}$ mice. Means for spleen weight and neutrophil, monocyte, lymphocyte, MPP2, MPP3, MPP4, MPP3/4, LT-HSC, ST-HSC, and CMP frequencies are displayed (A-K). *p≤0.05, **p≤0.01, ***p≤0.001, ****p≤0.0001. Error bars represent standard deviation. n=4-5 per group.

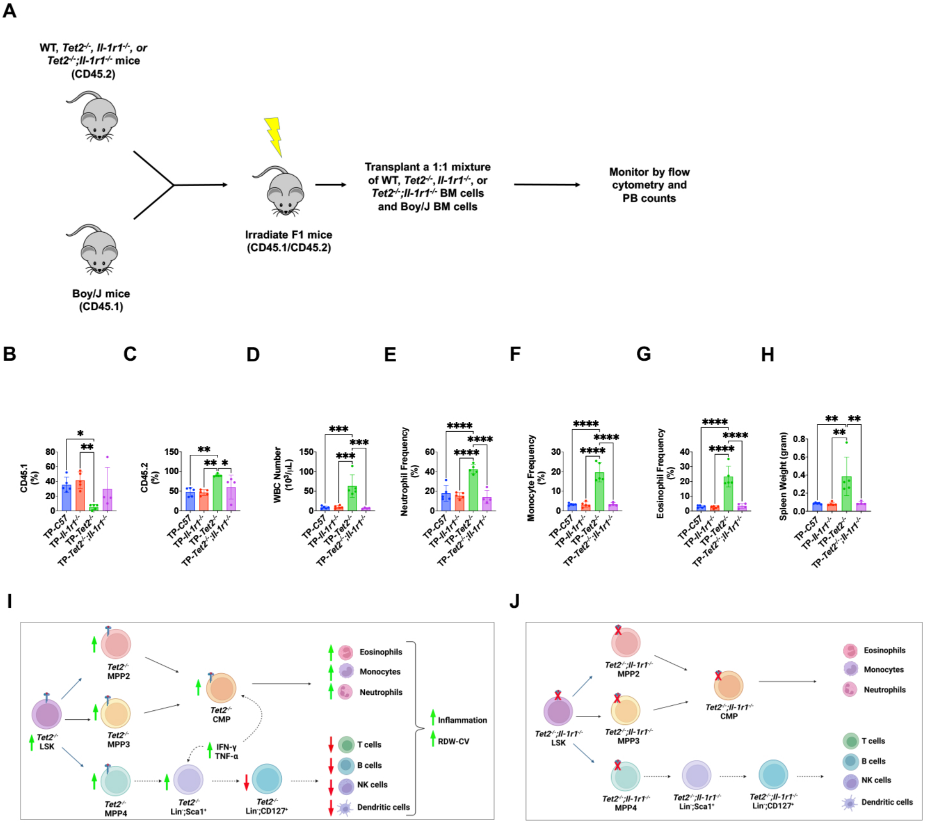

Supplementary Figure 2. CD45.2 engraftment, the levels of CD45.2-expressing myeloid cells, and spleen weight were rescued in mice transplanted with *Tet2-/-;Il-1r-/-* BM. A schematic of the transplant (TP) approach is shown (A). Mean frequencies for CD45.1 and CD45.2 expression in PB are displayed (B,C). Means of WBC number (D), the frequencies of neutrophils, monocytes, and eosinophils (E-G), and spleen weight at six-months post-transplant are presented (H). Schematics illustrate the putative mechanism by which loss of *Il-1r1* alleviates the disrupted myeloid-lymphoid shift and the pro-inflammatory state in *Tet2*-deficient HSPCs and were created with BioRender.com (I,J). *$p \leq 0.05$, **$p \leq 0.01$, ***$p \leq 0.001$, ****$p \leq 0.0001$. Error bars represent standard deviation. n=4-5 per group.

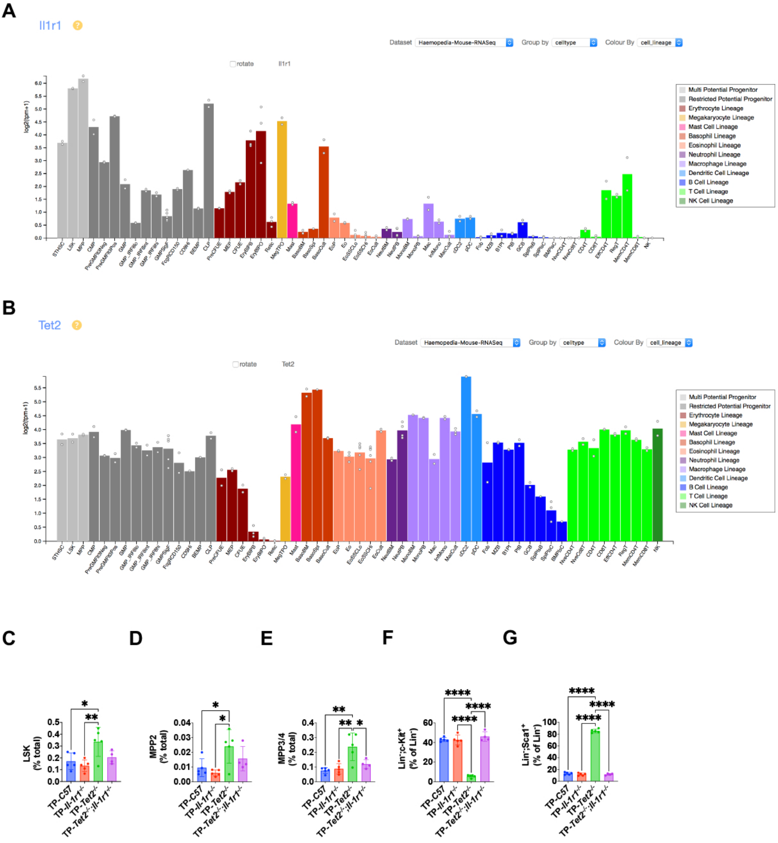

Supplementary Figure 3. *Il-1r1* and *Tet2* were strongly expressed in murine HSPCs, and loss of *Il-1r1* restored the levels of LSK cells and various immature hematopoietic populations. RNA expression for *Il-1r1* (A) and *Tet2* (B) from the Haemosphere database is shown [17]. Means of LSK (C), MPP2 (D), MPP3/4 (E), Lin$^-$;c-Kit$^+$ (F), and Lin$^-$;Sca1$^+$ (G) cells in mice transplanted with BM from *C57*, *Il-1r1*$^{-/-}$, *Tet2*$^{-/-}$, or *Tet2*$^{-/-}$;*Il-1r1*$^{-/-}$ mice are shown. *p≤0.05, **p≤0.01, ***p≤0.001, ****p≤0.0001. Error bars represent standard deviation. n=4-5 per group (C-G).

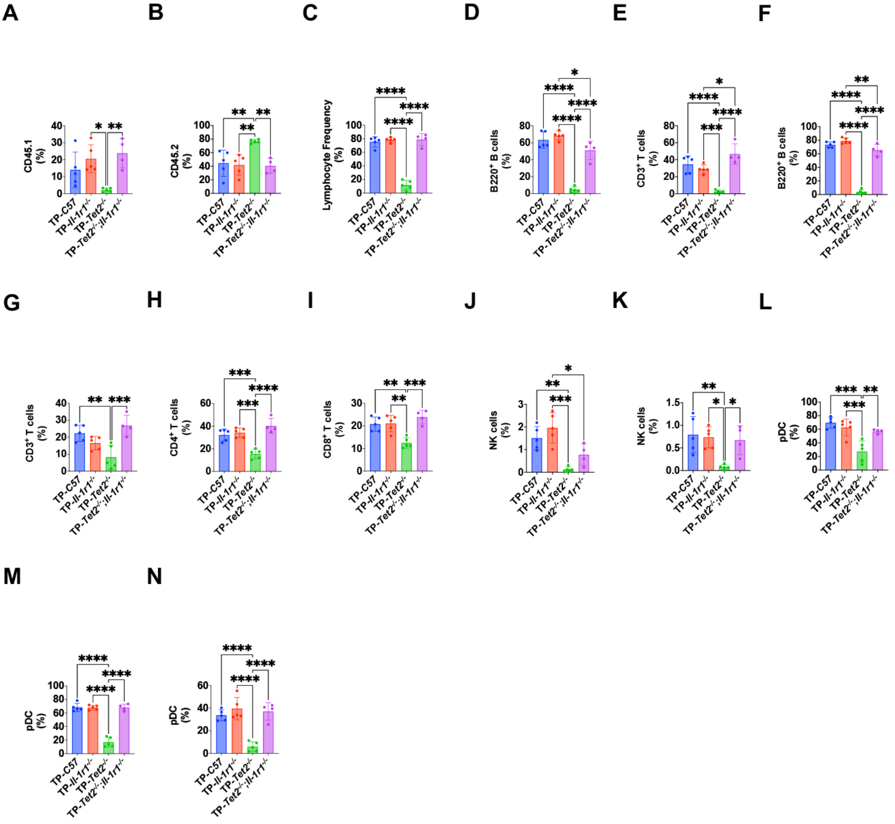

Supplementary Figure 4. Loss of *Il-1r1* corrected CD45.2 engraftment in the BM and alleviated suppression of multiple lymphoid cell types, including T cells, B cells, NK cells, and pDCs. Mean frequencies of CD45.1 and CD45.2 in the BM (A,B), lymphocytes in the PB (C), B cells and T cells in the PB (D, E) and spleen (F, G), CD4$^+$ and CD8$^+$ T cells in the spleen (H,I), NK cells in the PB and spleen (J,K), and pDCs in the PB, BM, and spleen (L, M, N) are shown. *p≤0.05, **p≤0.01, ***p≤0.001, ****p≤0.0001. Error bars represent standard deviation. n=4-5 per group.

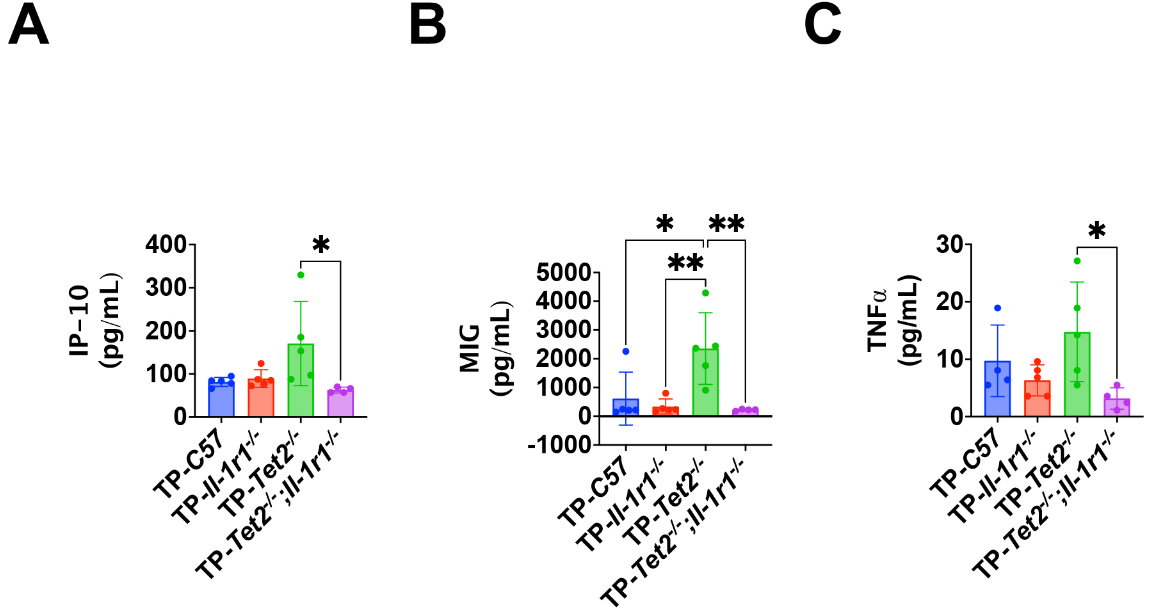

Supplementary Figure 5. Loss of *Il-1r1* rescued the levels of TNFα and IFN-γ-induced cytokines and chemokines. The levels of IP-10, MIG, and TNFα are shown (A-C). *p≤0.05, **p≤0.01, ***p≤0.001, ****p≤0.0001. Error bars represent standard deviation. n=4-5 per group.

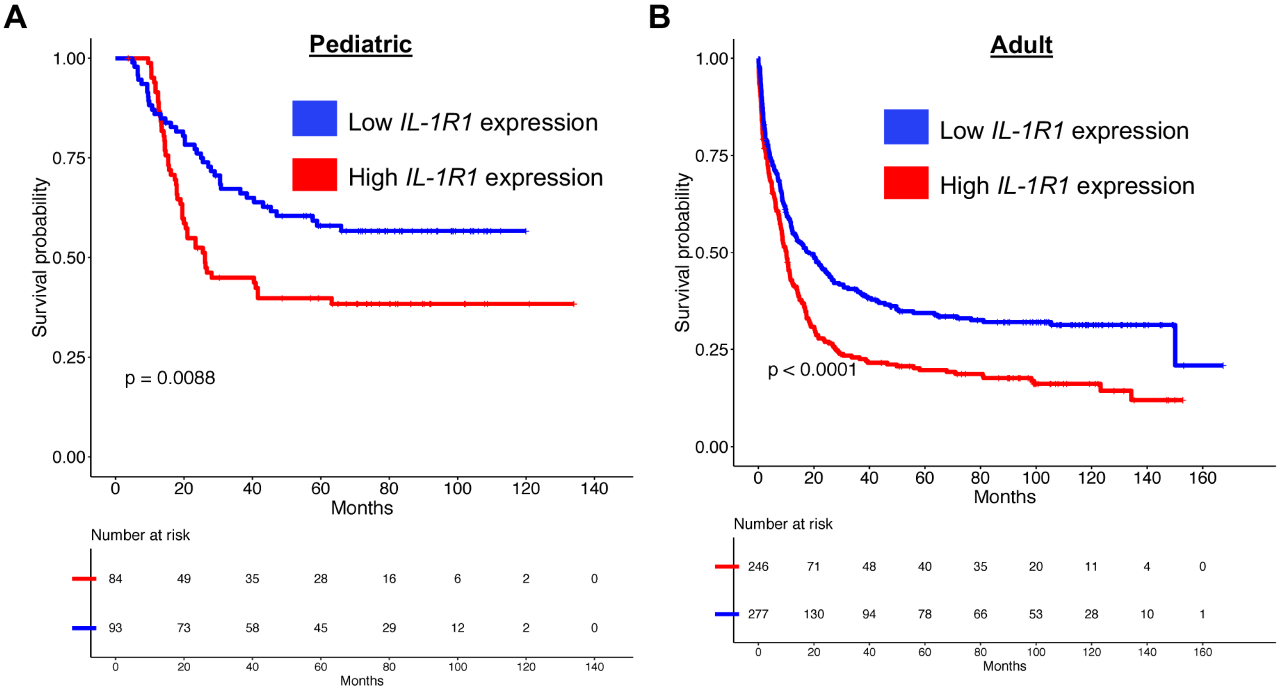

Supplementary Figure 6. *IL-1R1* expression correlated with decreased survival in pediatric and adult AML. Survival probability is displayed for pediatric (A) and adult (B) AML patients. The x-axis represents the survival time evaluated by month, and the y-axis shows the survival probability. Samples with high or low *IL-1R1* expression are shown in red and blue, respectively.

A

AML Subtype	Genetic Abnormalities
NPM1	AML with mutations in the NPM1 gene
TP53	AML with mutations in the TP53 gene
Splice	AML with mutations in genes involved in chromatin regulation, RNA splicing, or both
CBFB-MYH11	AML carrying inv(16)(p13.1q22) or t(16;16)(p13.1;q22)
PML-RARA	AML carrying t(15;17)(q22;q12)
MLL	AML carrying MLL fusion genes with multiple fusion partners for the MLL gene [t(x;11)(x;q23)]
CEBPA-Biallelic	AML with biallelic mutations in the CEBPA gene
RUNX1-RUNX1T1	AML carrying t(8;21)(q22;q22)
IDH^{R172}	AML carrying IDH2^{R172} mutations without other class-defining lesions
Inv3	AML carrying inv(3)(q21q26.2) or t(3;3)(q21;q26.2)

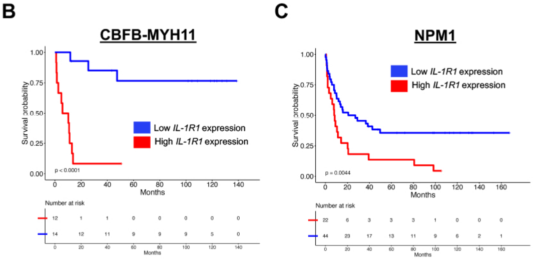

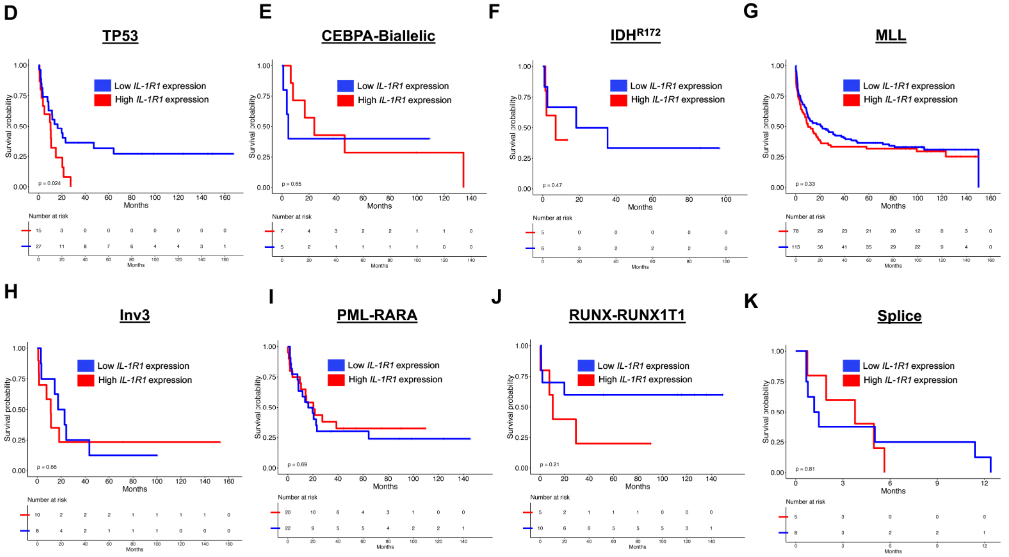

Supplementary Figure 7. Specific adult AML subtypes exhibited survival differences associated with the levels of *IL-1R1* expression. Ten AML subtypes were identified when stratified by subtype-specific genes (A). Survival probability is presented for each subtype (B-K). The x-axis represents the survival time evaluated by month, and the y-axis shows the survival probability. Samples with high or low *IL-1R1* expression are displayed in red and blue, respectively.
